# Risk and resilience on learning outcomes in diverse Muslim youth

**DOI:** 10.1371/journal.pmen.0000593

**Published:** 2026-04-02

**Authors:** Mehak Y. Shahzad, Tania M. Rodriguez, Rachel Wu

**Affiliations:** 1 Department of Clinical Psychology, Alliant University, Alhambra, California, United States of America; 2 Department of Psychology, University of California Riverside, Riverside, California, United States of America; PLOS: Public Library of Science, UNITED KINGDOM OF GREAT BRITAIN AND NORTHERN IRELAND

## Abstract

Muslim youths in the U.S. are facing mental health issues due to discrimination, bullying, and islamophobia, which may impact academic learning outcomes. However, there is considerable diversity in Muslim youth: the vast majority immigrating from or have parents from various geographic regions: Southeast Asia, Middle East, North Africa, Europe, etc. A few studies have reported group differences with discrimination regarding the Muslim population. Given the different cultural contexts and intersections of identities, more research is needed to better understand how the diversity of Muslim youth in the U.S. may require different tailored interventions and prevention programs that foster positive learning outcomes. This review paper starts by reviewing research reporting factors impacting the well-being of Muslim youth. Then, it highlights differences in experiences that may affect learning outcomes, such as geographic region, ethnicity, immigration status, minority status, and income level. It also discusses protective factors that may help buffer negative learning outcomes derived from experiencing discrimination, bullying, and islamophobia. Lastly, it provides suggestions for future research to help develop tailored interventions. A better understanding of how factors impacting well-being and learning outcomes differentially affect subgroups of Muslim youth, will allow for more nuanced, holistic understanding of an underserved, but growing population.

## Introduction

Almost 4 million Muslims reside in the United States [1]and it is anticipated that they will become the second-largest religious group in the country by 2040 [2]. However, they are an understudied population and there are only a handful of research papers that focus on the experiences of Muslim youth [3–5]As a religious group composed of multi-ethnic and cultural individuals that face prejudice and discrimination [6], it is essential to gain a comprehensive understanding of how Muslim youth are affected by anti-Muslim sentiments. Indeed, Muslims are considered one of the most discriminated groups, and instances of anti-Muslim discrimination have increased over the last few years [7–9]. With the current socio-political climate and anti-Muslim sentiments on the rise, it is important to address how Muslim youth are impacted.

There has been an increase in anti-Muslim sentiment under President Trump, leading to many Muslim Americans feeling ostracized and demonized by both the public and media [10]. With current policies (e.g., the Muslim Ban), the current political rhetoric and continued media portrayals of Muslims as security threats [11], it is no surprise that there was an increase in Muslims seeking mental health services for anxiety and stress related to concerns of personal safety [12]. Currently, 63% of Muslims continue to experience religious discrimination both in structural and social settings [12]. Muslim students, in particular, report feelings of fear related to surveillance and safety concerns [13]. The passing of the PATRIOT Act in 2001 has enabled surveillance of Muslim immigrants, American Muslims, and Islamic religious institutions [11]. In response, Muslim students reported the need to preemptively change their behavior in an effort to reduce possibilities of violence [13]. This is especially concerning given 47% of Muslim families currently have children in school who are facing religious based bullying [12]. Reflecting these trends, the National Islamophobia Index has increased from 25 in 2022–33 in 2025 [12].

In this review paper, we begin by covering the risk factors rooted in anti-Muslim sentiments that negatively impact the mental health and academic outcomes of Muslim adolescents and young adults, which include islamophobia, discrimination, and bullying. Next, we discuss the need for research to consider the diversity that exists within Muslim youth in the U.S. regarding geographic region, ethnicity, immigration status, minority status, and income level, given that differences across these factors could lead to distinct outcomes across youth. Lastly, we provide an overview of the protective factors that could help buffer the negative effects of the aforementioned risk factors. We conclude by making recommendations for future research to generate tailored interventions that benefit and help Muslim youth navigate anti-Muslim sentiments in society.

## Methods

We searched Google Scholar and the Ebsco Discovery Services search engine to find peer reviewed journal articles from relevant databases, including Springer, SAGE, Taylor & Francis, and more. To keep the cited literature as recent as possible, while also capturing the limited articles on Muslim youth in a wide enough time window, our search included articles from January 2000 to January 2026. Search terms included “Muslim youth,” “academic outcomes,” “America,” “bullying,” “Islamophobia,” “discrimination,” “resilience,” and similar variations and synonyms (academic achievement, protective factors, risk factors) were used in various combinations using both natural language search and with Boolean terms AND and OR. The inclusion criteria included any articles discussing the experiences of Muslim youth in schools, including Muslim college students, due to the scarcity of articles looking at the academic outcomes of Muslim youth from elementary to high school. A second wave of literature collection was conducted to include articles on Muslim youth's academic experiences and experiences with risk factors from other countries, given the limited number of articles specifically on American Muslim youth.

## Risk factors rooted in anti-muslim sentiment

Risk factors are defined as any biological, psychological, family, community, or cultural factors that are associated with a higher likelihood of adverse outcomes (e.g., substance abuse, poor mental health, and lower academic performance) [14]. Although a single risk factor might not always be a cause for concern, accumulating multiple risk factors increases the risk of problematic behaviors and adverse outcomes [15,16]. In regards to youth, some possible risk factors include low self-esteem, poor relationships with parents, peer rejection, disruptive family environment, and stressful or traumatic life events [14,17]. Additional risk factors commonly present among Muslim youth inside and outside of school settings include experiences of Islamophobia, discrimination, and bullying. These factors, when not mitigated by protective factors, can lead to risky behaviors such as smoking, drinking, drug use, and unsafe sexual practices [18]. Aside from risky behaviors, these risk factors can also lead to adverse outcomes such as higher psychological distress, lower academic aspirations, and lower well-being [16,19].

### Islamophobia

A prominent risk factor experienced by Muslims includes Islamophobia: a prejudice, hostility, or discriminatory attitudes and perceptions towards Muslims solely based on their religion. In schools, 1 in 4 Muslim students mentioned an adult had made offensive comments regarding Muslims or Islam [20]. However, it is not only limited to schools and can be found in classrooms, playgrounds, supermarkets, or neighborhoods [21]. It can lead to Muslim youth feeling emotions of sadness, alienation, isolation, and social marginalization [21]. Balkaya and colleagues [22] found that higher levels of Islamophobia were associated with Muslim youth feeling less connected with their American identity. Interestingly, in one study, young adult Muslims who were suspected or accused of wrongdoing were more likely to identify with their Muslim identity over their American identity [23]. Another study found that a weak Muslim and American identity was related to more internalizing and externalizing problems in Muslim youth [22]. Thus, Islamophobia can have serious consequences for Muslim youth beyond the classroom.

Within schools, Muslim students have experienced being called terrorists, being told to “go back to their country,” having their hijab ripped off in the hallway, and being glared at by their peers [5,19]. Besides peers, teachers and other authority figures can also be perpetrators of Islamophobia in school settings. It is crucial for educators to understand and respect the cultural and religious beliefs of Muslim students. Teachers and other staff members may commit microaggressions against Muslim students by alienating the culture and history of Islam through teaching inaccurate curriculum and not acknowledging the religious holidays and practices of the students [3,19,24,25]. This lack of cultural competence can lead to situations where students feel alienated and misunderstood. Cultural competence is a lifelong process and commitment to effectively working with various cultural communities. Individuals demonstrate an ability to be responsive to the needs of community members, accept differences, and work towards better understanding the differences [26]. It is important for educators to strive for cultural competence and create an environment where all students feel respected and valued.

For instance, in a qualitative research study investigating the experience of Muslim students in schools, a student reported that their physical education teacher refused to accommodate them since they were unable to swim due to modesty reasons associated with their culture [25]. The teacher then asked the student to run laps around the pool, resulting in the student feeling like she was being punished and humiliated for not participating in the swimming activity. In class, peers would repeatedly call Muslim students “Osama bin Laden” and ask if they had a bomb or were going to blow something up [20,27]. Muslim women wearing a hijab believe dressing modestly is related to lower perceived support from faculty and peers [24]. Additionally, Muslim students often feel compelled to represent Islam whenever related topics arise, with classmates eagerly awaiting their responses to gauge their stance on terrorism or other issues [28]. This pressure contributes to mental distress and anxiety, prompting some students to downplay their religious affiliation to alleviate the burden [18,28]. These instances of Islamophobia, where people automatically assume the worst of any person they perceive to look Muslim, should not be taken lightly, as they can have lasting negative impacts on Muslim students’ emotional well-being and academic outcomes.

### Discrimination

Alongside Islamophobia, another common risk factor that affects Muslim youth is discrimination, a broader version of Islamophobia that involves unfair treatment towards someone based on specific characteristics (including ethnicity), which leads to biases in people’s behaviors, decisions, and treatment towards that person. A survey showed that 75% of Muslims believe there is much discrimination in the U.S., and a staggering 48% reported experiencing at least one incident of discrimination within the past year [6].

In one instance, a teacher played the Muslim call to prayer as a prank while a student attempted to answer a question during a game, causing the entire class to laugh [29]. In another incident, a teacher told a student, “their type fly planes into buildings” [20]. Another involves a classmate saying, “Watch out, she has a bomb,” to get a laugh out of his friend at the expense of the Muslim student [29].

Discrimination has led to Muslim youth feeling unwelcome and uncomfortable at schools [5,19,30], impacting identity development [23], resulting in feelings of exclusion from the group [22], and high psychological distress [16]. This distress is not to be underestimated, as it can lead to an increased chance of engaging in risky behaviors [18] and was also seen to be correlated with more severe major depressive symptoms and generalized anxiety disorder symptoms for college students [31]. Some Muslim youth feel the need to participate in civic engagement activities to demonstrate they are “good citizens” [32]. These findings are also consistent in Muslim adults [33,34]. In other racial and ethnic groups, discrimination was associated with poorer academic performance and increased psychological maladjustment [35]. The negative consequences and impact of discrimination on the well-being of Muslim youth are clear and must be urgently addressed.

### Bullying

Bullying is another risk factor faced by many Muslim youth students in America, as they experience higher rates of bullying compared to the national average [36]. In California, 41.7% of Muslim students have reported being bullied, and 55.7% of students have felt unsafe or unwelcome in school [20], with 38% of incidents of bullying involving teachers or staff [37]. Students in school were verbally assaulted by being called “terrorists” and “sand n-word” or physically assaulted by pulling a Muslim girl’s hijab off [20]. Within the Muslim student population, research has suggested that bullying can lead to increased psychological distress, questioning of self-worth and self-esteem, as well as isolation, which can turn into depression or anxiety and have an overall negative effect on psychological well-being [18,29,36,37]. This heightened anxiety can stem from a place where students feel as if they are being targeted on purpose, which can result in difficulty concentrating or learning in class due to a low sense of belonging and a fear of safety [38,39]. With these risk factors, it is vital that we help develop strategies to improve the resiliency of this population and mitigate adverse outcomes. These outcomes include risky behaviors that can lead to mental health disorders and greater levels of substance abuse [14,15,40,41]. We are aware of protective factors that can aid in mitigating risk factors associated with bullying, but methods of implementing and raising awareness of them are still being developed.

### Intersections of risk factors

Although the risk factors mentioned above are spoken about in isolation, in reality individuals simultaneously experience multiple kinds of risk factors that compound on one another. Islamophobia can look increasingly complex when accounting for other identities an individual may have. Canadian and British Muslim women students experienced more Islamophobia than Muslim men [13,42–44]. Most likely due to Muslim women being easily identifiable when they dress Islamically through a hijab (headscarf), niqab (face veil), or abaya (full length outer garment) [13,44]. According to findings from Alizai [13], Muslim women experienced verbal harassment with obscene slurs and remarks such as “you terrorist go bomb other places.” When local authorities are unresponsive and fail to acknowledge hate crimes such as these it leads to Muslim women being hypervigilant, feeling less safe, excluded, and vulnerable [13,44]. Muslim women also felt increase pressure to represent Islam by watching what they say, mannerisms, and behavior. However, this constant self-monitoring leads to increased psychological burden for women [44]. This marginalization of Muslim women leads to them being on two extremes, either they are women who oppressed by religion (because of their dress choice), or they are seen as a threat because they are Muslim.

Not only is there a gendered difference in regards to Islamophobia, but other ethnicities and races also have different experiences with Islamophobia and discrimination. Black Muslim students in Britian experience racism that compounds with the experiences of discrimination [43]. Not only do the female Muslim students hear comments on their dress style, but they also face additional comments mocking and sexualizing their skin color and being compared to “charcoal” [43]. South Asian and other brown Muslims experience racism for their skin color as well [44]. It is not uncommon for Muslim students to be racialized and feel unsafe due to race over religion [45,46]. One Muslim American student mentioned how he tries to keep his ethnicity and religion private, so he can pass off as being white to avoid being discriminated against for his ethnicity, race, and religion [45]. Muslim students will be bullied, stereotyped, and experience overt racism all on top of religious discrimination [46].

Refugee and immigrant Muslims experience xenophobia, as they are seen as foreigners. Both within America and Canada these Muslims experience people calling them “terrorist” and saying that “this country should stop letting anyone in here” or they “can’t trust Muslims” which leads to feelings of exclusion and isolation [32,47]. They are not accepted as members of society and are expected to assimilate and change to fit the majority societal norms [47]. As they are adjusting to a new country and working towards overcoming a language barrier, refugee and immigrant Muslim youth, must also face racism, discrimination, and Islamophobia while they attend school too add to their burden [32].

We also acknowledge that Islamophobia intersects with many more identities and risk factors related to particular types of prejudice, including: anti-Brown, anti-Black, anti-Arab, and anti-Palestinian racism, xenophobia, homophobia, among other issues (see [44, 48–53] for more information on these topics). It is also important to note the sources that contribute to Islamophobia, including securitization, state surveillance, immigration policy, racialization, and public discourse around Muslims (see [54–62] for further information on these topics). Although an in depth review of both literatures is beyond the scope of this paper, other published work provides useful information for understanding and gaining insight into these nuanced and intricate topics.

## Diversity within the muslim population

In this section, we will discuss how the diversity of the Muslim population is represented in the current literature. We will describe diversity issues that are often present in studies conducted in the U.S., including ethnicity, race, immigration status, and geographic diversity.

While the cultural, ethnic, and racial diversity of the Muslim population has been recognized [18,27,36] in the literature, it is difficult to recruit a sample that mirrors the diversity of this population within the U.S. [36]. This can be a problem because it hurts the generalizability of research studies. It can be challenging to claim that the impact of a risk factor can be generalized to the broader Muslim population in America when the sample is overrepresenting a specific ethnic or racial group. However, it should be recognized that finding places to recruit Muslim participants can be challenging [63], especially if it is among adolescents where parental permission is required. This could be due to participants’ language barriers, lack of understanding of research, and mistrust of researchers collecting data [63,64]. Additionally, there is no existing central list of American Muslims as the U.S. census does not gather data on religion, which makes it challenging to select participants for a representative sample. These barriers for researchers make it increasingly challenging to gain a representative sample, resulting in the high prevalence of snowball or convenience sampling [3,29,65].

Besides focusing on risk factors for adverse mental health and behavioral outcomes among Muslim youth, the diversity of the population should also be taken into account when understanding the impacts and outcomes of the geographical, racial, and ethnic diversity of the Muslim population. There is no single country that makes up the majority of the U.S. Muslim population, nor is there a majority racial or ethnic group [1]. The largest racial group within the Muslim population is White (including Arabs, Middle Eastern, Persian, and Iranian) at 41%, followed by South Asians at 28%, and Blacks at 20% [1]. There is also a large immigrant population, with 58% of Muslims being first-generation Americans, 18% second-generation Americans, and 24% native-born Americans [1]. Considering these variations, it is important to understand how intersectionality may play a role in the experiences of Muslim youth as it pertains to the many different, multifaceted identities they carry.

One prevalent pattern in research papers is the overrepresentation of certain ethnic groups. Many studies have samples where middle-eastern American youth comprise the majority or entirety of the sample [23,36,65,66] or have a majority of South Asian participants [5,16,22]. While this is not inherently problematic, it becomes misleading when review papers about Muslim youth use findings from these studies and claim they apply to Muslims in general without clarifying the overrepresentation of a specific ethnic group. This oversight can lead readers to erroneously generalize the findings to any Muslim, disregarding the wide diversity of individuals within the Muslim population. Many studies also fail to consider the intersecting identities of Muslim individuals and how such intersections lead to unique experiences and perspectives. Therefore, it's crucial not to conflate ethnic groups with religion when interpreting results and to clearly state the limitations of studies with Muslim populations that compromise generalizability. Instead of striving to make findings sound generalizable when it's not the case, the focus should be on highlighting the diversity among Muslim individuals.

Intersectionality is a theoretical framework that highlights how the different identities that make up an individual combine to lead to unique experiences for each individual. The term, first coined by Dr. Kimberle Crenshaw [67], has historical roots in both a feminist and critical race theory framework, which originally represented the historical suffering of Black women in having to choose between fighting for their rights as women or as Black individuals [68]. The idea of voicing one’s concerns about both the racism and sexism faced by Black women was unheard of and outside the norm of society. Intersectionality analyzes how various identities, across levels, are awarded varying degrees of power and opportunity based upon the overlap of their identity statuses [68].

In this case, it is crucial to consider the unique experiences of different ethnic groups within the Muslim population. Nadal [27] found that microaggressions towards Muslims do not only occur because of religious affiliation but also stem from race, ethnicity, and gender. Combining these factors would lead to different discrimination experiences for different individuals. For example, a white female Muslim student will experience discrimination differently from an Asian male Muslim student. Rippy and Newman’s [34] study with Muslim adults showed that when a correlation between paranoia and discrimination was found, it was moderated by group differences among different ethnic populations. These findings imply that different ethnic groups within the Muslim population perceive and are affected by discrimination differently. As such, considering the Muslim population's diversity is important when conducting a study to make generalizable claims. Moreover, when working with Muslim youth, the intersectionality of identities during this developmental period may be more challenging to disentangle as they are figuring out the saliency of their identities. Therefore, researchers must be even more careful and conscious of potential differences when generalizing with Muslim youth.

## Learning outcomes among muslim youth

Investigating learning outcomes among Muslim youth is crucial as they tend to spend a significant amount of time in the school environment, and it has a significant impact on shaping their developmental trajectories. Prior literature has stated that low academic achievement can lead to negative externalizing behaviors such as defiance and aggression [15]. The fact that so few papers measure the impact of risk factors such as bullying, discrimination, and Islamophobia on learning outcomes in the Muslim youth population in America is problematic, given that we know there are many negative consequences that these risk factors can pose on academic success based on studies conducted with other populations. Racial and ethnic discrimination has been shown to lower academic self-efficacy, performance, and achievement among Black, Latino, and Asian populations [69–71]. Bullying was linked to poorer academic achievements and performance within other populations [72–74]. Understanding the impact of these risk factors on learning outcomes among Muslim youth is important, as it can help devise strategies to mitigate their effects and improve academic success.

While the majority of research on risk factors and learning outcomes is based on African-American, Latino, Asian, and other immigrant populations [69,71,73,75], some articles have focused on Muslim students. The consensus is that the aforementioned risk factors will harm academic performance, achievement, and engagement for Muslim students [3,18,30,37,76]. On college campuses, it has been seen that challenges stemming from risk factors can have both indirect and direct effects on the academic performance of students, highlighting the real-world implications of this research [18]. Hailu and colleagues [77] found having a culturally engaging and inclusive campus environment for Muslim college students is likely to be linked to better academic self-efficacy and performance. Muslim students who dealt with more daily hassles at school reported lower levels of educational aspirations [16]. Additionally, when dealing with prejudiced views and biased curricula portraying Islam in a negative light, students experience decreased academic performance and lower GPAs [37]. A study with a diverse sample of students from multiple high schools found that Muslim students of all ethnicities were equally likely to report higher academic achievement and expectations, but this was hindered by discrimination [16].

It is important to understand the outcomes of risk factors faced by this demographic. Because the majority of adolescence and early adulthood are spent in school, it is just as important to understand the impact on learning as it is to understand the mental health outcomes. Although there are few papers analyzing the effect of risk factors on Muslim American youth's learning outcomes, we can get a broad sense of its impact by looking at research done on other populations. Studies on bullying and peer victimization in elementary schools have shown negative correlations with academic achievement scores, academic functioning, and academic difficulties [39,78]. A further look into Arab American students supports prior findings of bullying and discrimination leading to worse academic outcomes [79]. Muslims in high school expressed having negative feelings towards school due to discrimination and bullying [32].

When expanding our outlook to Muslim youth in various other countries we have found that Muslim youth demonstrate increased educational resilience, in spite of discrimination faced, due to community and familial support [80]. Another study by Walker and Zuberi [47] found that discrimination is more likely to have a negative impact on academic ability and achievement. With students who are bullied, by non-Muslims, to have a stronger negative correlation with academic achievement [81]. Female Muslim college students in Canada felt a need to “outshine” their classmates to counter the assumption they are “academically inferior” [13]. British Muslim students had high academic achievement due to the high familial expectations of going to university, with Muslim girls outperforming Muslim boys regarding school performance [82].

## Resilience factors

The negative consequences of risk factors on a student's well-being and academic outcomes can be mitigated by the presence of protective factors. The link between these resilience and academic outcomes is well-supported by empirical research [3,16,33,37,76,83–85], and are further explored below.

Starting at the individual level—faith—a personal protective factor, allows youth to attribute their misfortunes to an external source, fostering a sense of hope and optimism and leading to better mental health outcomes [33,86]. Ahmed and Ezzeddine [87] found that higher religiosity leads to more significant character strengths (i.e., kindness, leadership, self-regulation, gratitude, optimism, and forgiveness), which can improve resiliency within the Muslim youth population. Within other religious groups, higher religiosity among adolescents was linked with better grades and less truancy [83]. Generally, having a strong sense of belonging to a group or strong identity has helped buffer the effects of the aforementioned risk factors and has shown to lead to low levels of psychological distress as well as less internalization and externalization of behaviors [3,16,22,37,76]. Stronger religious identity is also related to higher academic self-efficacy, higher academic performance, and fewer feelings of rejection [16,88].

At the school level, for academic success, it is crucial that educators play a significant role in making Muslim students feel welcomed and valued [89]. When teachers and staff allow students to practice their faith, support them, recognize religious holidays, and acknowledge their faith in the class curriculum, it helps them feel seen, safe, and welcomed [30,66]. Reducing prejudice in classrooms, allows youth to more actively engage in learning and academics, rather than be overwhelmed trying to “fit in” [30]. When youth feel supported by faculty and peers, they have better attendance which increases their problem-solving abilities and career opportunities [90]. Additionally, a culturally inclusive and engaging environment has been linked with better academic self-efficacy and performance for Muslim college students [77]. One study with Arab American students showed that a sense of belonging and affirmation positively correlated with GPA scores and scholastic competence [84]. Understanding the significance of these protective factors for Muslim students is essential for educators to develop and apply specific strategies to support their well-being and academic achievement.

Considering the multifaceted nature of protective factors, at a macro level, it has been recommended that positive family, peers, and community relations can help strengthen the mental health of Muslim youth [18,37,65]. This includes organizations such as MSA (Muslim Student Association) or youth-centered religious community activities, allowing students to feel safe and visible [18,37,76]. Increasing community level religious support for Muslim students was linked with fewer symptoms of major depression and generalized anxiety disorder [85]. Not only is community and family support important for mental health outcomes, but it can also help Muslim youth develop educational resilience [80]. It is crucial to view this population through an assets perspective, rather than a deficit lens. This approach empowers us to acknowledge the problem, but also to observe, find solutions, and test those potential solutions to see their effectiveness.

## Future directions

Overall, this paper highlights the mental health and academic outcomes of various risk factors for the Muslim youth population, including Islamophobia, discrimination, and bullying. Alongside the importance for future studies to take into account the ethnic and racial diversity of the Muslim population, as well as how many intersecting identities of Muslim youth may be influencing their experiences. Additionally, this study addressed the academic outcomes of the Muslim youth population and the lack of research studying this key area. Finally, we highlighted potential resilience factors clinicians and schools can consider when finding ways to help this population.

There are steps we can take to improve upon these problems in the literature. One such step is shifting from a deficit lens, focusing only on the risk factors, to a strengths-based approach that holds the potential to significantly improve the population's resilience. Another step is addressing steps that can be taken to help improve diversity when collecting samples for research projects.

### Need for more resiliency papers

A common theme throughout the literature is to focus mainly on the risk factors that affect Muslim youth. However, few papers study how to buffer or minimize the negative impact of such risk factors with the help of protective factors. Protective factors can be personal, such as a strong sense of identity, or environmental, such as a supportive family or community. This is concerning as we do not know if current resilience strategies implemented among other populations of youth will work similarly for Muslim youth or be less effective. Resiliency is essential in minority populations as it focuses on strengths and protective factors that can help protect an individual from stressors and adverse outcomes [91]. When dealing with bullying, discrimination, or other traumatic life events, protective factors can prevent individuals from falling into risky behaviors such as drugs and alcohol [18]. Due to insufficient papers directly studying this, it is difficult to make a recommendation or devise a strategy. Papers that mention protective factors tend to do so towards the end as a recommendation of possible methods to help.

### Addressing the lack of diversity

As mentioned earlier, it is crucial for future work to acknowledge the difficulty in gathering diverse samples and implement strategies that get us closer to achieving more representative samples of the diversity within the Muslim population. Many of the studies conducted with Muslim samples recruit participants from local mosques, convention centers, and schools, but this has resulted in sample sizes that are not diverse or large enough. This lack of diversity can lead to biased results and limit the generalizability of the findings. To address the lack of diversity, researchers need to develop trust with the community to help improve the chances of community members agreeing to participate [63]. Researchers could also look for participants in Islamic schools or MSA clubs that are present in middle schools through college. Additionally, large annual Islamic conventions like ISNA (Islamic Society of North America), MAS (Muslim American Society), or MSA West conferences have a large, diverse group of Muslims attending, so advertising could also be done there. All these societies also have social media pages, so it could be possible to ask if they would advertise flyers on their social media page to gain participants.

When studying diversity within the population, researchers can focus on specific subgroups, such as having a study focus specifically on Arab Muslims or South Asian Muslims rather than claiming a study is for the entire Muslim population when the sample is biased and over-represents a particular subgroup.

### Recommendations for future interventions

The risk factors Muslim youth face daily have significant negative impacts on their mental health. Aside from the prior resilience factors mentioned, therapy can be an additional support for this population. Although there are not many specific papers focusing on therapy recommendations for Muslim youth, there are a few papers with clinical recommendations for specific ethnic subgroups within the Muslim population.

When helping Arab American youth, Hashem and colleagues [92] recommended that therapists be aware of some aspects of Arab culture. Specifically, that family is an important source of support, as Arabs are a collectivistic culture. Additionally, honor is important to them. When seeking out therapists, they are more likely to seek out therapists of the same gender due to gender socialization rules within the culture. Importantly, this population expresses many mental health problems, such as anxiety and depression, as somatic complaints rather than verbalizing them, as is more common in Western culture. Alongside this, Arab youth face the difficulties of balancing two cultures and will need help with acculturation. When providing therapy for Arabs, clinicians should clarify it is a collaborative process that takes time as many young Arabs will see the therapist as an expert who will provide direct, immediate solutions.

Many of these recommendations for Arab American youth also hold for South Asian youth who also come from a collectivistic society and may share the same religion of Islam as the Arab youth. Sharma and colleagues [93] also mention that South Asian youth express mental health symptoms as somatic symptoms. This population also prefers “holistic” and “homeopathic” medicine over Western medicine, which is seen to have too many side effects. One other issue therapists working with South Asian youth face is that mental health issues are seen as moral failure, weakness, or due to supernatural causes within the culture [93]. This stigma against mental health makes it difficult for South Asian youth to seek help and open up to anyone, including their family. To combat this, clinicians can use a more medical and biological approach to explaining the mental illness to the client and family [93]. Providing psychoeducation and explaining mental health problems as medical problems allows for it not to be attributed to the youth’s personality or as a weakness in their character and decrease the stigma associated with therapy.

South Asian youth face acculturative stress from balancing two cultures, South Asian and American, and their different values [93]. Emphasis is placed on the importance of family, which can be included in the therapy process if the client wants. However, when problems arise due to differences in communication style and cultural values between parent and child due to acculturation, the therapist should work towards finding common ground between the two parties [93]. Both to help build a therapeutic alliance with the client and to help decrease stress felt by South Asian youth due to family conflict, when it is an important part of their culture and can act as a protective factor. An additional factor South Asian youth deal with that therapists should be aware of is the ‘model minority myth,’ which is the stereotype that all Asian Americans are high-achieving and academically successful, and its potential impacts, as they may feel overwhelmed due to academic pressure placed on them by family [93].

Aside from specific ethnic subgroups, there are a few qualitative papers in the literature with diverse and representative samples of the diversity within the Muslim population that outline some common themes and recommendations for working with Muslim clients. McLaughlin and colleagues [94] found that Muslims would like therapists of a similar background to them or of the same gender. Additionally, community-based treatments and outreach to Muslim communities to create a network of known therapy providers can decrease barriers to treatment and make mental health services more accessible to this population. Therapists must create a safe environment for Muslim clients, ensuring they have a positive experience with therapy and are more likely to return to therapy in the future or recommend it to others. This responsibility to create a safe space is a key part of a therapist's role with Muslim clients.

In Hodge and colleagues’ [95] paper, they found that Muslims want therapists who have a basic understanding of their beliefs and practices, as Islam is seen as a way of life and not just a religion. Many Muslims are afraid clinicians will have biases against them and would not respect their beliefs, rather imposing their values on the client. This belief can lead to the pathologizing of faith and blaming mental health issues on religion. Due to this, clinicians should work on any personal biases they may have towards Muslims and actively practice cultural competence to avoid disrespecting a client’s faith. Participants in the study also mentioned that they want clinicians to be aware of the ethnic and cultural differences amongst Muslims, as they are not a homogenous group.

## Conclusion

Having a more nuanced understanding of the impact of risk factors on Muslim youth is essential to aid in developing strategies to help increase resiliency within this population, especially given that it is made up of many different ethnic groups, and cultures. The majority of Muslim youth are in school, necessitating a greater number of studies to understand how their learning and academics are impacted by Islamophobia, bullying, and discrimination. Identifying risk and protective factors, as well as intervening in optimal ways, will improve resiliency for Muslim youth. The investment in their positive mental health and success would be highly beneficial not only for the individuals, but also for society in general.
